# Evaluation of Resistance Development to the Gwt1 Inhibitor Manogepix (APX001A) in *Candida* Species

**DOI:** 10.1128/AAC.01387-19

**Published:** 2019-12-20

**Authors:** Mili Kapoor, Molly Moloney, Quinlyn A. Soltow, Chris M. Pillar, Karen Joy Shaw

**Affiliations:** a Amplyx Pharmaceuticals, San Diego, California, USA; b Micromyx, Kalamazoo, Michigan, USA

**Keywords:** APX001, fosmanogepix, APX001A, manogepix, MGX, Gwt1, antifungal, *Candida*, resistance

## Abstract

Manogepix (MGX) targets the conserved fungal Gwt1 enzyme required for acylation of inositol early in the glycosylphosphatidylinositol biosynthesis pathway. The prodrug fosmanogepix is currently in clinical development for the treatment of invasive fungal infections. We determined that the median frequencies of spontaneous mutations conferring reduced susceptibility to MGX in Candida albicans, C. glabrata, and C. parapsilosis ranged from 3 × 10^−8^ to <1.85 × 10^−8^. Serial passage on agar identified mutants of C. albicans and C. parapsilosis with reduced susceptibility to MGX; however, this methodology did not result in C. glabrata mutants with reduced susceptibility. Similarly, serial passage in broth resulted in ≤2-fold changes in population MIC values for C. tropicalis, C. auris, and C. glabrata. A spontaneous V163A mutation in the Gwt1 protein of C. glabrata and a corresponding C. albicans heterozygous V162A mutant were obtained. A C. glabrata V163A Gwt1 mutant generated using CRISPR, along with V162A and V168A mutants expressed in C. albicans and Saccharomyces cerevisiae Gwt1, respectively, all demonstrated reduced susceptibility to MGX versus control strains, suggesting the importance of this valine residue to MGX binding across different species. Cross-resistance to the three major classes of antifungals was evaluated, but no changes in susceptibility to amphotericin B or caspofungin were observed in any mutant. No change was observed in fluconazole susceptibility, with the exception of a single non-Gwt1 mutant, where a 4-fold increase in the fluconazole MIC was observed. MGX demonstrated a relatively low potential for resistance development, consistent with other approved antifungal agents and those in clinical development.

## Abstract (precis)

Manogepix (MGX) targets the conserved fungal Gwt1 enzyme required for acylation of inositol early in the glycosylphosphatidylinositol biosynthesis pathway. The prodrug fosmanogepix is currently in clinical development for the treatment of invasive fungal infections.

## INTRODUCTION

Fungal diseases continue to be a significant cause of human morbidity and mortality, with invasive fungal infections (IFIs) estimated to kill more than 1.5 million people worldwide annually (1, 2). Patients who are immunocompromised are especially vulnerable to these infections (3). Treatment of IFIs is limited to approved agents in three drug classes: azoles, polyenes, and echinocandins, with the latter being the only new class of antifungal agents developed in the last 30 years. Limited treatment options that are safe and well tolerated, along with the growing emergence of resistance to currently available antifungals, have resulted in an urgent need for the discovery and development of new agents.

The glycosylphosphatidylinositol (GPI) biosynthesis pathway is an essential cellular process for many eukaryotes and has emerged as an attractive target for novel antifungals. This pathway is required to anchor mannoproteins to the cell wall of fungi (4). These mannoproteins have critical functions, including providing structural integrity to the cell wall, adhesion of pathogenic fungi to mucosal surfaces, and facilitating replication of pathogenic fungi at mucosal surfaces that can result in a disseminated infection (5–7). The Gwt1 enzyme catalyzes the third step of the GPI anchor biosynthesis pathway, which involves acylation of inositol. When Gwt1 is blocked either genetically or chemically, the structural integrity of the cell wall is compromised, inhibiting fungal cell growth of *Aspergillus* and *Candida* (8).

Fosmanogepix (APX001, formerly E1211) is a first-in-class antifungal agent that is currently in clinical development for the treatment of invasive fungal infections (9, 10). Fosmanogepix is an N-phosphonooxymethyl prodrug, which is rapidly and completely metabolized by systemic phosphatases to the active moiety, manogepix (MGX; formerly APX001A and E1210) (8). MGX inhibits the fungal Gwt1 enzyme (4, 11), resulting in pleiotropic effects on the fungal cell due to inhibition of cell wall mannoprotein localization, as well as compromised cell wall integrity, biofilm formation, germ tube formation, and fungal growth (12, 13). MGX does not inhibit PIGW, the closest mammalian ortholog of the fungal Gwt1 protein (12). This impressive selectivity is ascribed to both the low (<30%) homology between Gwt1 and PIGW and the diverse functions of these proteins in humans and fungi (13).

MGX has broad *in vitro* activity against major fungal pathogens, including *Candida*, *Cryptococcus*, *Aspergillus*, *Scedosporium*, and *Fusarium* species (14–18) and retains activity against azole-resistant and echinocandin-resistant strains of *Candida* and *Aspergillus* both *in vitro* and *in vivo* (19, 20). No dose-limiting toxicities were observed when fosmanogepix was administered to healthy volunteers at exposures that are expected to translate to clinical efficacy against the major fungal pathogens (9, 10). These drug characteristics suggest that fosmanogepix has potential as a treatment option for patients with life-threatening invasive fungal infections.

In this study, we evaluated the potential for *Candida* species to develop resistance to MGX by analyzing both spontaneous mutation frequencies and resistance development by serial passage. These species included C. albicans, C. glabrata, C. parapsilosis, C. tropicalis, and C. auris*. Candida* mutants were evaluated for cross-resistance to other classes of antifungal agents, as well as gepinacin, a structurally unrelated Gwt1 inhibitor (13).

(Portions of this work were presented at 2017 ASM Microbe [M. Kapoor, M. Moloney, Q. Soltow, and K. J. Shaw, poster 296, San Diego, CA].)

## RESULTS

### Resistance development by spontaneous mutation.

**(i) Susceptibility.** The MIC of MGX was determined for three *Candida* ATCC strains (C. albicans 90028, C. glabrata 2001, and C. parapsilosis 22019) using the Clinical and Laboratory Standard Institute (CLSI) M27-A3 broth microdilution method and reading of the MIC at 50% growth inhibition relative to the growth control (21). The MGX MIC value for both C. albicans and C. parapsilosis was 0.016 μg/ml, and that for C. glabrata was 0.03 μg/ml. The MIC value of amphotericin B (AMB) for C. albicans (read at 100% growth inhibition) was 0.5 μg/ml.

The broth MIC values were used to determine the ranges of concentrations for agar MIC assays using Sabouraud dextrose agar (SDA) plates. The final agar MIC values were the same for C. albicans, 2-fold higher for C. parapsilosis, or 4-fold higher for C. glabrata than the corresponding broth MIC value for each strain. The agar MIC value for AMB was the same as its broth MIC value of 0.5 μg/ml.

**(ii) Frequency of spontaneous mutations.** The spontaneous frequency of resistance to MGX was determined in triplicate on agar at 4× MIC for the three *Candida* strains using 245-by-245-mm square bioassay dishes, as previously described (22, 23). After incubation, all putative mutant colonies were confirmed after repeat streaking on plates containing MGX. Spontaneous mutation frequencies were calculated by dividing the number of resistant colonies on a given plate by the plating CFU. Median spontaneous mutation frequencies for MGX ranged from 3 × 10^−8^ to <1.85 × 10^−8^ (Table 1). The frequency of resistance to AMB at 2× MIC using C. albicans 90028 ranged from 5.15 × 10^−8^ to 8.78 × 10^−9^.

**TABLE 1:** MGX spontaneous mutation frequencies

Strain	Replicate plate no.	No. of colonies	Resistance frequency at 4× MIC
*C. albicans* 90028	1	3	3.00 × 10^–8^
	2	20	4.08 × 10^–7^
	3	2	1.72 × 10^–8^
*C. glabrata* 2001	1	0	<1.88 × 10^–8^
	2	4	2.29 × 10^–8^
	3	0	<1.52 × 10^–8^
*C. parapsilosis* 22019	1	2	1.31 × 10^–8^
	2	0	<1.85 × 10^–8^
	3	3	6.20 × 10^–8^

### Resistance development by serial passage.

**FIG 1:**
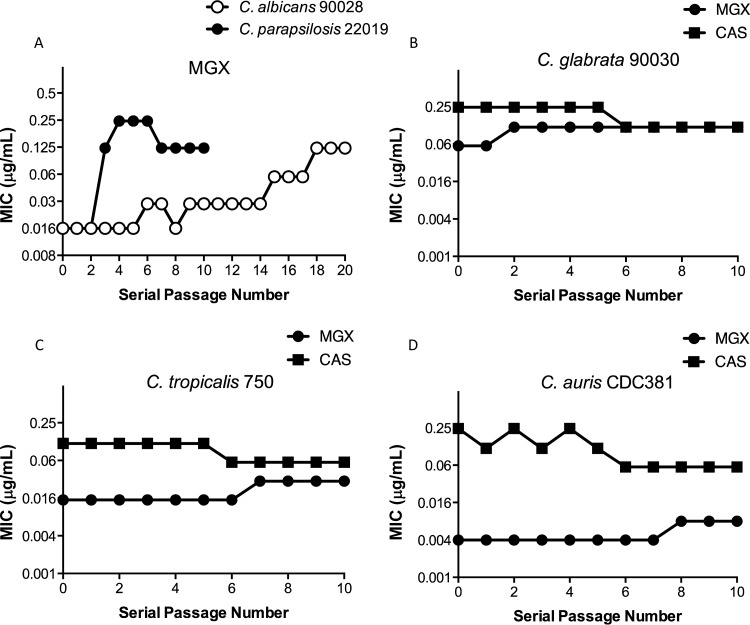
(A) Gradient plate serial passage log_2_ MIC plots for MGX versus C. albicans 90028 and C. parapsilosis 22019 populations. (B to D) Broth macrodilution serial passage total population log_2_ MIC plots for MGX and CAS versus C. glabrata 90030 (B), C. tropicalis 750 (C), and C. auris CDC381 (D). Plots show the total population MIC values versus the serial passage number.

**(i) Gradient plate method.** SDA plates containing MGX concentration gradients were prepared as previously described using 90-by-90-mm square petri dishes (22–24). The concentration of MGX was increased 2-fold in subsequent passages if growth exceeded the half-way point on the plates. MIC testing was performed on total cell populations for each passage. Individual colonies were obtained from selected populations and assessed for MIC, followed by sequencing the *GWT1* gene from the strain. Using this methodology, mutants were selected by plating large numbers of cells (10^6^) across a wide range of drug concentrations. The MIC of the total population versus the serial passage number is shown in Fig. 1A. For C. albicans 90028, MIC values increased 8-fold from 0.016 to 0.125 μg/ml after 18 serial passages. The total population MIC increased 8- to 16-fold for C. parapsilosis 22019 after three to four serial passages. Serial passage experiments were typically performed for 10 passages. However, only a 2-fold increase in MIC was seen at passage 10 (P10) for C. albicans, and therefore serial passages were continued until P20. Putative mutants were colony purified from P10 (C. parapsilosis) and P20 (C. albicans). MIC values were determined for each colony purified mutant.

**(ii) Broth macrodilution serial passage method.** Despite repeated attempts to serially passage C. glabrata 2001 using the gradient plate method, no changes in the MIC of the population were observed after 10 serial passages. In order to rule out the possibility that this was due to the agar plate-based methodology, broth macrodilution serial passage methods were used for C. glabrata 90030. In addition, C. tropicalis 750 and C. auris CDC381 were included in this method. The MIC values observed during 10 serial passages are shown in Fig. 1. The MIC values of MGX did not increase substantially (≤2-fold) during serial passage for these three *Candida* isolates. Caspofungin (CAS) was included as a comparator during the broth macrodilution serial passage, and no increase in CAS MIC was observed for the evaluated *Candida* isolates (Fig. 1).

### Characterization of mutant strains. (i) Determination of MIC values.

MGX broth MIC values were determined for a subset of mutants isolated from both the spontaneous mutant selection and the serial passage experiments using the CLSI M27-A3 broth microdilution method (21). As shown in Table 2, six C. albicans mutants demonstrated a 4-fold (*n* = 4), 8-fold (*n* = 1), and 16-fold (*n* = 1) increase in MIC compared to the wild-type (WT) strain. Four *C. glabrata* mutants demonstrated a 32-fold increase in the MGX MIC. Eight *C. parapsilosis* mutants demonstrated an 8-fold (*n* = 3) and 16-fold (*n* = 5) increase in the MGX MIC (Table 2).

**TABLE 2:** Susceptibilities of mutant and WT strains to MGX

Background	Derivation	Strain	MIC (μg/ml)	MGX MIC ratio (fold change over WT)	Gwt1 amino acid sequence
*C. albicans* 90028	WT		0.016		WT
	Spontaneous	5.1	0.06	4	WT
		5.2	0.03	2	WT
		5.3	0.06	4	WT
	Serial passage	4.15	0.25	16	V162A (heterozygous)
		P20-1	0.125	8	WT
		P20-2	0.06	4	WT
		P20-3	0.06	4	WT
*C. glabrata* 2001	WT		0.03		WT
	Spontaneous	5.1	1	32	V163A
		5.2	1	32	V163A
		5.3	1	32	V163A
		5.4	1	32	V163A
	CRISPR	RNP1	1	32	V163A
*C. parapsilosis* 22019	WT		0.016		WT
	Spontaneous	5.2	0.125	8	WT
		5.3	0.25	16	WT
		5.4	0.25	16	WT
		5.5	0.125	8	WT
	Serial passage	P10-1	0.125	8	WT
		P10-2	0.25	16	WT
		P10-3	0.25	16	WT
		P10-4	0.25	16	WT

**FIG 2:**
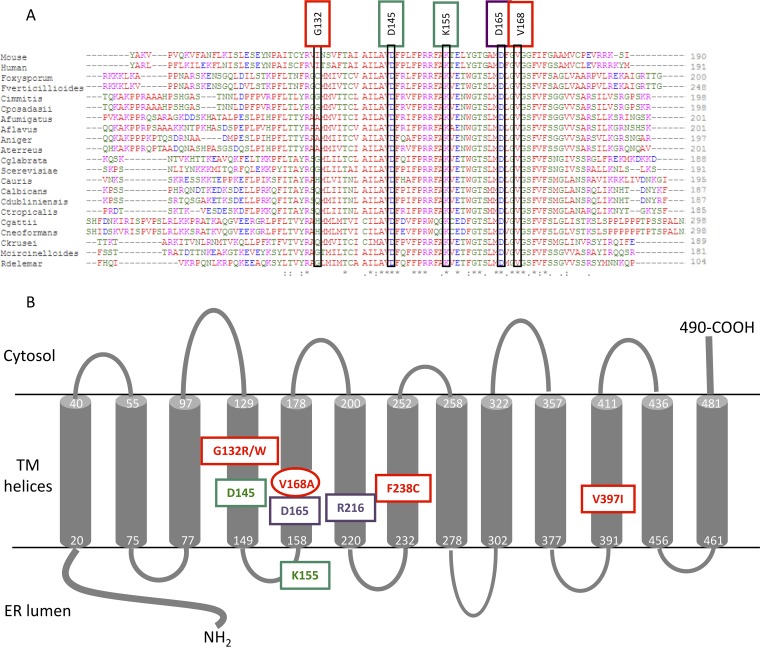
(A) Multiple sequence alignment of Gwt1 orthologs. (B) Putative location of important residues in the Gwt1 protein. The alignment shows a conserved region containing amino acid residues that have been shown to be essential for the activity of the fungal Gwt1 enzyme (D145 and K155 shown in green) and mammalian PIGW (D165 shown in blue), as well as those that lead to resistance to Gwt1 inhibitors (G132R/W shown in red) (30). The position of the V168 residue identified in mutants with elevated MIC values in this study (corresponding to C. albicans V162A and C. glabrata V163A) is also shown in red. Similar to G132R and G132W, the V168A mutation is predicted to lie within a transmembrane portion of the protein, rather than facing the lumen of the endoplasmic reticulum (30). Abbreviations: genus/species names are combined (e.g., Foxysporum is Fusarium oxysporum). Symbols beneath the sequence alignment are: asterisk (*), 100% sequence identity; colon (:), conservation between amino acids with strongly similar properties; period (.), conservation between groups of weakly similar properties. The figure is based on Sagane et al. (30), with numbering based upon the S. cerevisiae protein sequence. Residues essential for fungal Gwt1 activity (green), residues essential for human PIGW activity (purple), and residues that lead to resistance to Gwt1 inhibitors (red) (4, 25, 30; this study) are indicated.

**(ii) Identification of mechanisms of resistance.** To understand the underlying resistance mechanisms, *GWT1*, the gene encoding the MGX target protein, was sequenced in strains demonstrating an MIC increase of ≥2-fold and compared to the *GWT1* sequence from the respective starting WT strains. The primers used for DNA sequencing are shown in Table S1 in the supplemental material. A valine-to-alanine mutation at position 163 (V163A) in the Gwt1 protein was identified in four C. glabrata mutants (Table 2). Since these four strains were isolated from the same spontaneous mutation experiment, it is possible that the strains originated from a single event. The corresponding amino acid substitution V162A was found in a C. albicans heterozygous mutant arising in a serial passage experiment. This residue corresponds to V168 in S. cerevisiae Gwt1 (ScGwt1) protein sequence (Fig. 2). No other mutations in *GWT1* were detected among all other mutants analyzed.

Since C. glabrata has a haploid genome and non-*GWT1* mutations may be more readily identified, whole-genome sequencing of a nonsusceptible C. glabrata strain (C. glabrata 5.2, Table 2) was performed. This strain has 32-fold-higher MIC compared to WT C. glabrata. The genome was analyzed for single nucleotide polymorphisms (SNPs), insertions, and deletions with respect to the reference WT parent genome. In all, 10 nonsynonymous variants were obtained in 9 unique genes. Based on the annotations of the S. cerevisiae orthologs (shown in parenthesis), four genes (CAGL0C00847g [*FLO10*], CAGL0I06160g [*CIS3*], CAGL0L05852g [*NUP49*], and CAGL0M02343g [*ATG5*]) perform functions in biogenesis of cellular components, and four genes (CAGL0L03432g [*GWT1*], CAGL0L05852g [*NUP49*], CAGL0L09823g [*CDC16*], and CAGL0M02343g [*ATG5*]) perform functions in protein folding, modification, and destination. Two of the genes (CAGL0C00847g [*FLO10*] and CAGL0J05159g) code for proteins that are GPI anchored and are putative adhesin-like proteins. Further studies are needed to determine whether these mutations are causal and to delineate the possible roles played by these mutations toward elevations in MGX MIC values.

**(iii) Confirmation of resistance mechanism.** To confirm that the V163A mutation in Gwt1 led to reduced susceptibility of the C. glabrata strains to MGX, we created a C. glabrata strain with V163A Gwt1 using CRISPR and compared MGX MIC values versus the WT and mutant C. glabrata strain. The CRISPR V163A Gwt1 mutant strain demonstrated a 32-fold-higher MGX MIC compared to the parent WT strain (Table 2), the same as the 32-fold increase in MIC of the C. glabrata strain identified in the spontaneous mutant selection experiment. These data confirm that the C. glabrata V163A Gwt1 mutation is necessary and sufficient for the reduced susceptibility phenotype.

**(iv) Cross-resistance.** Cross-resistance to all three major classes of antifungals (AMB, fluconazole [FLC], and CAS) was evaluated for five mutants of the three species, representing both *GWT1* and non-*GWT1* mutants (Table 3). No changes in the MIC value of AMB or CAS were observed. In most of the mutants, no changes were observed in the MIC value of FLC. In a single non-*GWT1* C. parapsilosis mutant (5.2, Table 3), a 4-fold increase in the FLC MIC value was observed; however, no change in posaconazole or voriconazole MIC values was observed (data not shown).

**TABLE 3:** Evaluation of *Candida* mutants with decreased susceptibility to MGX for cross-resistance to other antifungals^*a*^

Background	Strain	Gwt1 amino acid sequence	MIC ratio (fold change over WT)			
			MGX	AMB	FLC	CAS
*C. albicans* 90028	5.3	WT/WT	4	1	2	1
	4.15	V162A/WT	16	1	1	1
*C. glabrata* 2001	5.2	V163A	32	1	1	1
*C. parapsilosis* 22019	5.2	WT/WT	8	1	4	1
	5.4	WT/WT	16	1	1	1

a Abbreviations: WT, wild type; MGX, manogepix; AMB, amphotericin B; FLC, fluconazole; CAS, caspofungin.

The Gwt1 and non-Gwt1 mutants were also evaluated for cross-resistance to gepinacin, an unrelated molecule that targets fungal Gwt1 (25). The V162A C. albicans and the V163A C. glabrata mutants demonstrated >16-fold and >4-fold increases in gepinacin MIC values, similar to 16- to 32-fold increases in MGX MIC observed for these strains. In addition, the non-Gwt1 C. albicans and C. parapsilosis mutants demonstrated a 2- to 4-fold increase in the gepinacin MIC values, similar to 2- to 8-fold increases in the MGX MIC values for these strains.

**(v) Susceptibility of *S. cerevisiae* strains expressing Gwt1 orthologs from a high-copy-number plasmid.** The Gwt1 human ortholog, PIGW protein was previously expressed from a high-copy-number 2-μm vector, under the control of the strong GAPDH (glyceraldehyde-3-phosphate dehydrogenase) promoter (YEp352GAPII) in an S. cerevisiae *gwt1Δ* background (YEp-human) in which the host is dependent upon the cloned heterologous gene for survival (12). MGX did not inhibit inositol acyltransferase enzymatic assay using membranes prepared from this strain (12). We observed a >250-fold increase in MGX MIC values for the YEp-human strain compared to the S. cerevisiae strains expressing *ScGWT1* from the chromosome (W303-1B) or from another 2-μm vector with a strong promoter (pSF-TEF1-LEU2, *ScGWT1*) alone (Table 4). In contrast, the MGX MIC values for S. cerevisiae-Gwt1 (ScGwt1) disrupted strains expressing WT and V162A C. albicans Gwt1 (CaGwt1) were 0.06 and 4 μg/ml, respectively, demonstrating a 64-fold increase in MIC value (Table 4). Similarly, the MGX MIC values for ScGwt1 disrupted strains expressing WT and V168A ScGwt1 increased 8-fold (0.06 and 0.5 μg/ml, respectively). These data suggest the importance of valine 168 in different Gwt1 orthologs.

**TABLE 4:** MGX susceptibility of S. cerevisiae strains expressing WT and mutant *GWT1* orthologs^*a*^

Strain	*GWT1* source	MGX MIC^*b*^ (μg/ml)
W303-1B	Chromosomal *S. cerevisiae* (WT)	0.03
ScGwt1-WT	pSF-TEF1-LEU2, *ScGWT1* (WT)	0.06
ScGwt1-V168A	pSF-TEF1-LEU2, *ScGWT1* V168A	0.5
CaGwt1-WT	pSF-TEF1-LEU2, *CaGWT1* (WT)	0.06
CaGwt1-V162A	pSF-TEF1-LEU2, *CaGWT1* V162A	4
CkGwt1-WT	pSF-TEF1-LEU2, *CkGWT1* (WT)	0.125
CnGwt1-WT	pSF-TEF1-LEU2, *CnGWT1* (WT)	0.125
AfGwt1-WT	pSF-TEF1-LEU2, *AfGwt1* (WT)	0.5
KE249	YEp352GAPII, human PIGW	>16

a All strains shown except W3031B have the same genotype. Genotypes: W3031B (*MATα ade2-1 his3-11 leu2-3*,*112 trp1-1 ura3-1 can1-100*); ScGwt1-WT (*MATα ade2-1 his3-11 leu2-3*,*112 trp1-1 ura3-1 can1-100 gwt1*::*his5^+^* [pSF-ScGwt1]); KE249 (*MATα ade2-1 his3-11 leu2-3*,*112 trp1-1 ura3-1 can1-100 gwt1*::*his5^+^* [YEp-hum]). Abbreviations: SD, synthetically defined; *Sc*, S. cerevisiae; *Ca*, C. albicans*; Ck*, C. krusei; *Cn*, C. neoformans; *Af*, A. fumigatus.

b The MGX MIC was determined in SD-LEU medium to maintain plasmid selection, except for the MGX MICs for W303-1B and KE249, which were determined in SD+LEU and SD-URA media, respectively.

Although MGX is highly active against *Candida* spp. (MIC_90_ ≤ 0.06 μg/ml), it demonstrates poor activity against Candida krusei (MIC_90_ ≥ 16 μg/ml) (15, 17). To help elucidate the underlying mechanism of C. krusei nonsusceptibility to MGX, we expressed the C. krusei Gwt1 protein (CkGwt1) in the S. cerevisiae *gwt1Δ* background and compared it to other similarly expressed fungal Gwt1 proteins (Table 4). The MIC value of the strain expressing the CkGwt1 protein (0.125 μg/ml) was 2-fold higher than isogenic strains expressing the C. albicans or S. cerevisiae WT proteins (0.06 μg/ml), suggesting that unlike expression of the human PIGW protein, which resulted in a ≥250-fold increase in MIC (Table 4), MGX inhibited the activity of the CkGwt1 protein and that C. krusei most likely has other non-target-based mechanisms of resistance to MGX.

## DISCUSSION

Resistance to all classes of antifungal agents has been previously observed, and the underlying mechanisms have been studied in great detail (26). In *Candida* spp. mutations or alterations of target Erg11, upregulation of multidrug transporters, and cellular stress-based responses lead to resistance to the azoles. Loss-of-function mutations in ergosterol biosynthesis genes and cellular stress responses lead to resistance to the polyenes. Mutations in the *FKS* genes, which encode the catalytic subunits of glucan synthase, are associated with reduced susceptibility to echinocandins (27). In addition, cellular stress responses can cause resistance to the echinocandins (28).

In this study, we evaluated the development of resistance to MGX in several species of *Candida* using both spontaneous and serial passage methods. Spontaneous mutations leading to elevated MGX MIC values in *Candida* spp. occurred at low frequencies, with median frequencies (3 × 10^−8^ to <1.85 × 10^−8^) in the range of those reported for other antifungals such as the echinocandins (1.59 × 10^−7^ to <3.86 10^−9^) (22).

A V163A mutation in Gwt1 was identified in C. glabrata strains with reduced MGX susceptibility. The corresponding V162A substitution was identified as a heterozygous mutation in a C. albicans strain, suggesting the importance of this residue in different orthologs despite the former strain being haploid and the latter strain being diploid. This amino acid residue (V168 using S. cerevisiae numbering) is located in a highly conserved region of Gwt1, a multiple membrane-spanning protein (Fig. 2). Alignment of fungal Gwt1 and mammalian PIGW protein sequences was performed using Clustal Omega analysis (29). The portion of the alignment shown in Fig. 2A highlights a region previously identified as critical for both fungal and mammalian enzyme activity (30). Sagane et al. demonstrated the presence of four highly conserved regions of the protein in luminal loops or near the luminal face of transmembrane domains (30). V168 lies in one such region that also contains the essential amino acid residues aspartic acid at position 145 (D145) and lysine at position 155 (K155) near the luminal face of transmembrane domain 4 and in loop-connecting helices 4 and 5, respectively. D145 and K155 are in close proximity to valine 168, present in helix 5, suggesting that the region is important for Gwt1 function, as well as the binding of MGX. In addition, mutations conferring resistance to other Gwt-targeting small molecules lie close to this region. A G132R mutant was resistant to BIQ, a previous-generation Gwt1 inhibitor (4), and a G132W mutant was resistant to G884, a structurally different Gwt1-targeting small molecule (25). Our experiments demonstrating reduced susceptibility of the V163A Gwt1 C. glabrata strain generated by CRISPR and S. cerevisiae strains expressing mutant Gwt1 proteins from a multicopy plasmid further suggest the importance of this residue toward MGX binding in different orthologs.

Previous studies overexpressed C. albicans and A. fumigatus Gwt1 genes, as well as the human PIGW gene, in a haploid *gwt1* disruption strain of S. cerevisiae, where growth was dependent upon the cloned gene (12). The plasmids had a 2-μm origin of replication (ori) and GAPDH promoter-driven expression of the Gwt1 proteins. Isolated membranes from these strains were evaluated in an inositol acylation assay for inhibition by MGX. The 50% inhibitory concentration values were 0.27, 0.6, and >36 μM for the C. albicans, A. fumigatus, and human enzymes, respectively. The MIC values for the three S. cerevisiae strains were 0.03, 0.5, and >32 μg/ml, respectively, consistent with the idea that MGX inhibits the fungal Gwt1 enzymes but not the human enzyme (12). In addition, MGX appeared to be less active against A. fumigatus enzyme by both enzymatic (2-fold) and microbiological (16-fold) measurements. In the present study, we expressed A. fumigatus, C. albicans, and S. cerevisiae Gwt1 using a plasmid with 2-μm ori and the TEF1 promoter. We also observed that the S. cerevisiae dependent upon the A. fumigatus protein is 8-fold less susceptible to MGX than the same strain expressing the S. cerevisiae or C. albicans Gwt1 WT proteins and similar to the isogenic S. cerevisiae expressing the ScGwt1 V168A mutant protein (Table 4). These data are consistent with the biochemical evidence that MGX is less active against the A. fumigatus protein (12).

Expression of the C. krusei and Cryptococcus neoformans Gwt1 proteins resulted in only a 2-fold increase in the MIC value versus expression of the S. cerevisiae and C. albicans Gwt1 proteins in an isogenic background (Table 4). These data suggest that the large increase in MIC values seen for these two organisms (C. krusei, MIC range, 2 to >32 μg/ml [15]; C. neoformans H99, MIC = 0.25 μg/ml [31]) versus C. albicans (MIC_90_ ≤ 0.008 μg/ml [15]) may not be due to differences in the target protein sequences *per se* but may be dependent upon other factors, such as permeability and efflux in the more resistant species.

Gepinacin belongs to another class of GPI synthesis inhibitors targeting fungal Gwt1 (25). Evaluation of the V162A C. albicans and the V163A C. glabrata mutants demonstrated >16-fold and >8-fold increases in gepinacin MICs, respectively, suggesting a binding site that is similar or overlapping with the MGX binding site. Interestingly, the C. albicans and C. parapsilosis strains with elevated MIC values but with no Gwt1 mutations were also less susceptible to gepinacin. These data suggest that, along with mutations in the Gwt1 target, other mutations such as those within the GPI anchor biosynthesis pathway or common uptake- or efflux-based mechanisms can potentially lead to resistance to Gwt1 inhibitors.

Previous studies had shown that MGX MIC values of *Candida* and MEC values of *Aspergillus* were unchanged in the presence of mutations leading to resistance to the echinocandins, itraconazole, FLC, and AMB (16, 17). Miyazaki et al. evaluated a collection of FLC-resistant (MIC ≥ 64 μg/ml and FLC-susceptible (MIC ≤ 32 μg/ml) *Candida* isolates and determined that the MGX MIC_90_ values were similar (0.03 and 0.06 μg/ml, respectively) (15), suggesting a lack of cross-resistance. More recently, MGX was tested against 100 geographically distinct C. auris isolates, and no correlation was observed between FLC and MGX MICs (32). In the present study, cross-resistance to antifungals belonging to the three major antifungal classes was largely not observed. Of the 19 mutants evaluated across *Candida* spp., we observed 2- to 32-fold increases in MGX MIC values over the WT strains. No change was observed in FLC susceptibility, with the exception of a single C. parapsilosis mutant where a 4-fold increase in the FLC MIC was observed concomitant with an 8-fold increase in the MGX MIC. No change in posaconazole and voriconazole MICs was observed for this strain versus the WT C. parapsilosis. Also, the strain did not harbor any mutations in *GWT1.* Arendrup et al. observed a correlation between MGX and FLC MIC values in a number of *Candida* species in a study investigating MGX susceptibility against 540 candidemia and 122 C. auris isolates (33). Excluding C. krusei, which has been previously shown to be largely resistant to MGX (15, 17), only 4 of 631 strains of *Candida* demonstrated MIC values above the WT upper limit (33). These strains included one C. dubliniensis strain (MIC = 0.03 μg/ml), two C. glabrata strains (MIC = 0.25 μg/ml), and one C. tropicalis strain (MIC = 0.125 μg/ml). These four strains had FLC MIC values of ≥16 μg/ml (33). This correlation was not observed in Candida guilliermondii and C. auris. The basis for the observed correlation to FLC reduced susceptibility in a subset of *Candida* strains is unknown but could potentially be due to changes in membrane composition and drug permeability, altered efflux, or other factors.

Increases were observed in the MGX MIC in serial passage experiments, which ranged from 2-fold in broth selection experiments to 16-fold in agar selection experiments. Given the low MICs of the starting strains, it may be possible that some of these MICs remain in the clinically treatable range. In addition, the phenotype of strains with reduced susceptibility obtained in the course of these experiments also suggested a loss in fitness of the strains. This was observed as certain strains growing poorly in broth or having smaller colony size compared to respective WT strains (data not shown). Clinical studies currently in progress may help to discern the impact of higher-MIC strains on treatment success.

Manogepix demonstrated a low potential for resistance development, consistent with other approved antifungals and a low potential for cross-resistance to other antifungal classes. Manogepix may offer a much needed new class in the limited treatment armamentarium for invasive fungal infections.

## MATERIALS AND METHODS

### Isolates tested.

Representative strains for C. albicans (ATCC 90028), C. glabrata (ATCC 2001 and ATCC 90030), C. parapsilosis (ATCC 22019), and C. tropicalis (ATCC 750) were obtained from American Type Culture Collection (ATCC; Manassas, VA), and C. auris (CDC381) was obtained from the Centers for Disease Control and Prevention (CDC; Atlanta, GA). S. cerevisiae strains KE666, KE249, and W303-1B were obtained from K. Hata (Eisai Co., Ltd.) (12).

### Antifungal agents.

The following antifungals were used in the study: AMB (VWR, Radnor, PA), FLC (Alfa Aesar, Tewksbury, MA), CAS (Sigma, St. Louis, MO), and MGX (Amplyx Pharmaceuticals). All drug stocks were prepared at 10 mg/ml in 100% dimethyl sulfoxide (DMSO), and aliquots were stored at −20°C.

### Antifungal susceptibility testing.

Drug susceptibility tests were performed using a broth microdilution method according to CLSI M27-A3 and read at 50% growth inhibition (21). Antifungals were first diluted in DMSO to obtain intermediate dilutions. These were further diluted in microtiter plates to obtain final concentrations of 16 to 0.008 μg/ml. A 1-μl aliquot of DMSO was added to “no-drug” control wells. The solutions were mixed by shaking on a plate shaker for 10 min, and plates were incubated at 35°C for 40 to 48 h. The minimum concentration that led to 50% reduction in fungal growth compared to the control was determined as the MIC.

The broth MIC values were used to determine the drug concentration ranges used to pour a series of SDA plates in order to determine the agar MIC values (i.e., the minimum concentration of each drug that would prevent the growth of macroscopically visible colonies of each species). The MIC values of S. cerevisiae strains expressing Gwt1 were determined similar to the CLSI methodology with some modifications. MIC values were determined in SD-LEU media to maintain plasmid selection, except for W303-1B and KE249 strains, which were determined in SD+LEU and SD-URA media, respectively. MIC plates were incubated at 30°C for 48 h.

### Spontaneous mutation analysis.

Spontaneous mutation frequencies for MGX were determined in triplicate for C. albicans, C. glabrata, and C. parapsilosis, using a previously described large-plate format method (22, 23). Assay dishes (245 by 245 mm; Corning) were prepared with 150 ml of SDA containing MGX at 4× agar MIC for each *Candida* strain. Aliquots (1 ml containing approximately 1 × 10^8^ CFU) were spread onto SDA plates containing MGX, followed by incubation at 35°C for 72 h. After incubation, putative mutant colonies were confirmed by streaking on plates containing drug. For each inoculum, plating CFU were determined, which ranged from 4.8 × 10^7^ to 1.9 × 10^8^. Spontaneous mutation frequencies were calculated by dividing the number of resistant colonies on a given plate by the starting viable count as determined by plating CFU. Glycerol stocks of putative mutant colonies were stored at –80°C and strains evaluated for changes in MIC and sequencing of *GWT1* gene.

### Serial passage.

Serial passage experiments were performed on gradient plates, as well as by broth macrodilution methodology. SDA drug gradient plates were prepared by pouring two overlapping layers of media as previously described (22, 23). Briefly, the bottom layer containing 25 ml of drug-free SDA was poured in 90-by-90-mm square petri dishes while on an incline. Once solidified, the plate was placed flat and the top layer was poured containing MGX at the minimal concentration that inhibited the growth of each strain fully but allowed for some growth past the edge of the plate containing no drug into the start of the drug gradient. After each passage, the leading edge of growth (i.e., cells growing at the highest concentration) was resuspended in 0.85% NaCl, and the yeast cells were counted using a hemocytometer. An aliquot of 1.0 × 10^6^ cells was spread onto a fresh passage plate. When the growth of strains with reduced susceptibility was observed at or past the halfway point of the gradient plate, drug concentrations were increased 2-fold for subsequent passages. A glycerol stock was made from the total cell population for each culture condition for each passage and the population MIC was determined. Total population MIC data were plotted using Prism (GraphPad Software, Inc.).

Serial passage by broth macrodilution was performed at Micromyx (Kalamazoo, MI). Twofold dilution series of MGX and CAS were made in RPMI with DMSO at a final concentration of 1% (vol/vol). For each concentration evaluated, a 2-ml volume was dispensed into polystyrene test tubes, leaving an additional tube without drug to serve as the growth control. For the initial inoculation (passage 0), colonies from freshly streaked agar plates were used to make a 0.5 McFarland standard equivalent for each test organism per CLSI methods (21), which was further diluted in RPMI to obtain a final cell density of approximately 0.5 to 2.5 × 10^3^ CFU/ml, followed by incubation for 22 to 24 h. After incubation, the MIC was recorded as the lowest concentration that significantly inhibited growth of the test organism (>50%) relative to the growth control. The MIC at passage 0 represented the activity of MGX and CAS against the test isolates prior to passage of isolates in the presence of subinhibitory concentrations of drug. For all subsequent passages, the inoculum for each evaluated drug series, and each organism was made directly from the tube below the MIC (the last tube exhibiting growth in the assay). The growth of the tube was adjusted to a 0.5 McFarland, diluted 1:100 in RPMI, and was used to inoculate a fresh set of tubes for the indicated drug series. If there was insufficient growth to achieve a 0.5 McFarland from the tube one dilution below the MIC, the growth from the tube one dilution below the MIC was combined with the tube two dilutions below the MIC. Inoculated tubes were incubated, and the MIC was read as described above. A 0.5-ml aliquot of the 0.5 McFarland suspension used at each pass was frozen after adding 20% glycerol as cryoprotectant to allow for subsequent analysis. The passages were conducted on consecutive days during the course of the experiment. The 0.5 McFarland suspensions used at passages 0, 3, 7, and 10 were quantitated to ensure that counts were approximately 1 × 10^6^ to 5 × 10^6^ CFU/ml for the suspension. C. parapsilosis ATCC 22019 was used as the control strain for broth microdilution testing, and the MIC values against this strain were unchanged throughout serial passage for both test agents (MGX, 0.008 to 0.015 μg/ml; CAS, 0.25 to 0.5 μg/ml), and the CAS MIC values were within the quality control range set for broth microdilution testing for this organism.

Serial passage on gradient plates was performed twice for the C. albicans 90028 and C. glabrata 2001 strains and once for the C. parapsilosis 22019 strain. Serial passage by broth macrodilution was performed once each for the C. glabrata 90030, C. tropicalis 750, and C. auris CDC381 strains.

### DNA sequencing.

*GWT1*, the gene coding for the MGX target protein, was sequenced in strains demonstrating increased MIC values and compared to *GWT1* sequence in the respective WT strains. Primers were designed to sequence ∼2 kb of DNA corresponding to ∼1,500 bases of GWT1 open reading frame and additional upstream and downstream regions (see Table S1 in the supplemental material). Separate primer sets were designed for each of the three *Candida* spp. To design the primer sets, *GWT1* reference sequences from C. albicans MYA2876, C. glabrata 2001, and C. parapsilosis 4646 were used. Genomic DNA was isolated from yeast colonies, and PCR was performed using the primers listed, followed by Sanger sequencing of the PCR products (Genewiz, South Plainfield, NJ). The DNA sequences obtained were compared to respective WT sequences using DNASTAR software (Madison, WI).

### Whole-genome sequencing and analysis.

Genomic DNA was isolated from C. glabrata 2001 WT and the 5.2 strain with reduced MGX susceptibility, followed by whole-genome sequencing using an Illumina MiSeq platform (Genewiz). During data analysis, sequence reads for the two samples were trimmed of nucleotides with poor quality, and their adapters were removed. The reads were next aligned to the reference C. glabrata 2001 sequence using CLC Genomics Workbench 9.0.1. SNPs/indels were detected using the basic variant detection model within the CLC Genomics Workbench (minimum frequency = 35%; minimum coverage = 10; minimum count of a variant = 4), followed by extraction of unique variants between the WT and mutant sample.

### CRISPR-based mutagenesis.

CRISPR-based mutagenesis was performed using ribonucleoproteins (RNPs) to generate a C. glabrata strain with a valine-to-alanine mutation at position 163 in Gwt1 using previously described methods (34). All transformations were performed by electroporation of competent cells prepared using lithium acetate (35). RNPs were created using the Alt-R CRISPR-Cas9 system (Integrated DNA Technologies, Inc., Coralville, IA). The CRISPR machinery included purified Cas9 protein and two RNAs: the CRISPR guide RNA (crRNA), gene specific for C. glabrata *GWT1*, and a universal transactivating CRISPR RNA (tracrRNA), which forms an RNA duplex with the gene-specific crRNA and subsequently complexes with the Cas9 nuclease. To ensure that the protein and RNA components are properly assembled and transformed together, the crRNA and tracrRNA were coincubated and then added to purified Cas9 protein, allowing formation of the RNA-protein complex prior to electroporation. The RNP complex was electroporated into C. glabrata 2001, along with single-stranded oligonucleotide (ssODN) containing the V-to-A mutation and a PAM site. After recovery, cells were pelleted (3,000 rpm, 3 min) and resuspended in 200 μl of yeast extract-peptone-dextrose (YPD) broth before aliquots were spread onto YPD plates, followed by incubation at 30°C for 2 days. To allow for selection of colonies containing the Gwt1 mutation, transformants were patched onto plates containing 0.5 and 1 μg/ml MGX. One colony was obtained on each of the two MGX-containing plates. Sequencing confirmed the presence of a mutation in *GWT1*. Sequencing also confirmed the presence of other silent mutations in *GWT1* as a result of ssODN template design, confirming that the mutants were indeed obtained due to CRISPR and not a result of a spontaneous mutation event. Negative-control transformation mixtures contained 40 μl of cell slurry and 10 μl of ice-cold 1 M sorbitol (no RNPs, no DNA repair construct). Negative controls did not yield any colonies when plated on YPD plus MGX but yielded robust growth on YPD alone.

### Plasmid-borne expression of Gwt1 orthologs.

The 2-μm vector pSF-TEF1-LEU2 (catalog number OGS531; Sigma-Aldrich) was used for cloning *GWT1* orthologs from C. albicans, C. krusei, C. neoformans, and S. cerevisiae. This plasmid contains the constitutive TEF1 promoter (translation elongation factor 1), along with a LEU^+^ selectable marker. Whole *GWT1* genes were synthesized as follows: C. krusei (GenBank accession no. OUT21717.1) and C. albicans (GenBank BAC66174.1), with silent mutations in the *GWT1* coding sequence to remove the NcoI restriction sites; C. neoformans (GenBank AFR96919.1), with intron sequences removed; and S. cerevisiae (GenBank DAA08709.1), with silent mutations in the coding sequence to remove NcoI and XbaI restriction sites. DNA sequences were added to the 5′ ends of the genes coding for the hemagglutinin tag at the N terminus of the expressed protein, a location hypothesized to face the lumen of the endoplasmic reticulum (30). The plasmids were transformed into the haploid S. cerevisiae KE666 strain (*MATα ade2-1 his3-11 leu2-3*,*112 trp1-1 ura3-1 can1-100 gwt1*::*his5^+^* [YEp-Ca]), in which the plasmid-borne C. albicans *GWT1* gene (under the control of the S. cerevisiae GAPDH promoter [36]) complements the chromosomal S. cerevisiae *gwt1*::*his5*^+^ mutation, thus providing the sole source of Gwt1 enzyme activity (37). To replace the existing YEp-Ca plasmid with the newly cloned pSF-TEF1-LEU2 vectors, yeast transformants were selected on leucine-dropout plates (gain of the pSF-TEF1-LEU2 vectors carrying *GWT1* homologs), followed by reverse selection on 5-fluoroorotic acid (loss of YEp-Ca URA3). To ensure that the original plasmid carrying the C. albicans *GWT1* plasmid had been lost, final clones were confirmed by both uracil auxotrophy and PCR amplification to contain only the LEU^+^ plasmid carrying the heterologously expressed fungal *GWT1*.

## Supplementary Material

Supplemental file 1
