# Supplementary material for: Evaluation of Resistance Development to the Gwt1 Inhibitor Manogepix (APX001A) in Candida Species
Source: Antimicrob Agents Chemother. 2019 Dec 20;64(1):e01387-19. doi: 10.1128/AAC.01387-19 (PMC7187586; doi:10.1128/AAC.01387-19)
Supplement: Supplemental file 1 [file AAC.01387-19-s0001.pdf]

SUPPLEMENTARY INFORMATION

Table S1. Primers used for sequencing *GWT1* from *Candida* strains obtained in spontaneous and serial passage experiments

Species	Primer Name	Primer Sequence
C. albicans	MYA2876-1F	GTAAAGAATCGAATTGACAACGG
C. albicans	MYA2876-1R	GGTAACAACTCAATTATGCGGGG
C. albicans	MYA2876-2F	CCAAACTGTTCACTGAACAACC
C. albicans	MYA2876-2R	GGATGCTAGAATTGTCAACAC
C. albicans	MYA2876-3Fnew	GCTGTAACCAGTATAGCATTATC
C. albicans	MYA2876-3R	GGCACTGACTTGATAAAGTTCTGC
C. albicans	MYA2876-4F	CCCAAGAAGATTTGCCAAAG
C. albicans	MYA2876-4R	GTTCTTTGTTTTGTAGCCTG
C. albicans	MYA2876-5F	GAAGTTCATTTTGAGCAGCG
C. albicans	MYA2876-5R	CACATCTATGGTATGATACC
C. glabrata	glabrata-F	CGTTAACATTAGCAATGTCGG
C. glabrata	glabrata-R	CACACTCTTCCGCAATCAAG
C. glabrata	glabrata-SeqFl	TAACTGGGTTGCTATGCTTC
C. glabrata	glabrata-SeqF2	TAGTCTATTAACCTACTTGG
C. glabrata	glabrata-SeqR1	ATCCAATGGTTTGTCCCCAT
C. glabrata	glabrata-SeqR2	ATAGTTGAGAAGAAACCACC
C. parapsilosis	MYA-4646-F	ATCCAATGAGGTTGATGTGG
C. parapsilosis	MYA-4646-R	GTTCACAGTAAAACACTCG
C. parapsilosis	MYA-4646-SeqF1	ACTATACAAGCAAAAAGAGG
C. parapsilosis	MYA-4646-SeqF2	ATCGTTTGGATCGTTTGTCC
C. parapsilosis	MYA-4646-SeqRl	AAAATGAGTATATCCCTTCC

C. parapsilosis	MYA-4646-SeqR2	GCAAAAACATGAGTAGTGATGG
C. parapsilosis	MYA-4646-SeqF	CCATCAACAAAAGATAGCTG
